# Integrative Transcriptomic and Proteomic Analysis Reveals *CaMK4*-Mediated Regulation of Proliferation in Goat Skeletal Muscle Satellite Cells

**DOI:** 10.3390/ani15213083

**Published:** 2025-10-24

**Authors:** He Cong, Lu Xu, Yaolong Liu, Zixuan Wang, Tao Ren, Pengcheng Ruan, Haoyuan Zhang, Chengli Liu, Yanguo Han, Pengfei Hu, Yan Zeng, Simone Ceccobelli, Guangxin E

**Affiliations:** 1 College of Animal Science and Technology, Southwest University, Chongqing 400715, China; 2 Institute of Antler Science and Product Technology, Changchun Sci-Tech University, Changchun 130022, China; 3 Department of Agricultural, Food and Environmental Sciences, Università Politecnica Delle Marche, Via Brecce Bianche 10, 60131 Ancona, Italy

**Keywords:** *CaMK4*, skeletal muscle satellite cells (MuSCs), cell proliferation, transcriptomics and proteomics

## Abstract

**Simple Summary:**

Calcium/calmodulin-dependent protein kinase IV (*CaMK4*) is involved in a variety of biological processes in skeletal muscle satellite cells (MuSCs). In this study, we found that *CaMK4* has a inhibitory effect on the proliferation of MuSCs in goats, and clarified that *CaMK4* overexpression or knockdown significantly regulates a series of candidate genes related to muscle development and cell proliferation in goats at both the transcriptional and protein levels, which provides new mechanistic insights into genetic regulation of muscle development in goats.

**Abstract:**

*CaMK4*, a calcium/calmodulin-dependent protein kinase, is an important mediator of cellular signal transduction, yet its role in the regulation of skeletal muscle satellite cells (MuSCs) in goats has remained unclear. In this study, *CaMK4* overexpression and knockdown models were established, and integrated transcriptomic and proteomic analyses were performed to systematically elucidate its regulatory network. *CaMK4* overexpression altered key pathways associated with cell proliferation and muscle development, including cAMP, PI3K-Akt, and actin cytoskeleton regulation, while proteomic data highlighted calcium signaling and JAK-STAT pathways. Conversely, *CaMK4* knockdown enhanced MuSC proliferation by upregulating cell cycle-related genes and proteins. Integrated analyses further identified that Galectin-9 (*LGALS9)*, Collagen triple helix repeat containing-1 (*CTHRC1)*, Hyaluronan Synthase 1 (*HAS1)*, and L-Threonine Dehydrogenase (*TDH)* may serve as potential key nodes regulating cell cycle, apoptosis, and metabolic control. This suggests a regulatory role for *CaMK4*. Collectively, these findings provide a mechanistic framework for understanding *CaMK4* function in ruminant muscle development and may offer insights for improving goat muscle growth, meat quality traits, and production efficiency.

## 1. Introduction

Meat-type goats are a globally important economic livestock species, providing high-quality animal protein crucial for food security and nutritional supply worldwide. With the continuous growth of the global population and improvement of living standards, meat consumption has steadily increased. Goat meat, characterized by low fat and high protein content, has become a preferred healthy meat option, leading to a significant rise in demand. Particularly in developing countries, the goat industry has become an indispensable component of agricultural economies, contributing positively to rural development and farmers’ income growth [1,2]. In recent years, the rapid increase in global goat meat production highlights its critical role in meeting human meat-food demand. However, ensuring a stable supply not only requires expanding breeding scale but more importantly depends on understanding the genetic basis of key genes influencing meat production traits. This knowledge will underpin future genetic breeding strategies aimed at developing efficient and sustainable meat-producing livestock and pastoral industries.

Meat production in goats directly affects farming efficiency and industry sustainability. Skeletal muscle, the main meat component, is influenced by muscle fiber number and diameter, which determine muscle size and growth rate, while intramuscular fat affects flavor and metabolism [3,4]. Studies show a positive correlation between fiber diameter and muscle growth from birth to nine months, promoting rapid development [5,6]. Thus, understanding the molecular regulation of skeletal muscle is vital for improving meat yield and quality.

Muscle satellite cells (MuSCs) are the primary stem cell population responsible for adult skeletal muscle regeneration and growth. They reside between the muscle fiber sarcolemma and the extracellular matrix (ECM), comprising approximately 2% to 10% of total skeletal muscle cells, and exhibit high heterogeneity [7,8,9]. MuSCs provide new cellular sources for muscle fibers through proliferation and differentiation, directly influencing muscle repair and growth. Animal model studies demonstrate that depletion of MuSCs leads to loss of muscle regenerative capacity, underscoring their essential role in muscle development [10,11,12]. Therefore, regulating MuSC proliferation and differentiation is critical for enhancing skeletal muscle growth rate and quality.

Calcium ions (Ca^2+^), as vital intracellular second messengers, play central roles in regulating diverse cellular functions, particularly in muscle cell signaling and functional maintenance. The calcium/calmodulin-dependent protein kinases (CaMKs) family acts as key effectors of Ca^2+^ signaling pathways, modulating cell cycle progression, metabolism, and gene expression [13,14]. *CaMK4* has garnered attention for its potential role in skeletal muscle development. Predominantly expressed in the brain, immune, and reproductive tissues, *CaMK4* activity is regulated by the calcium/calmodulin complex and phosphorylates nuclear transcription factors such as the cAMP response element-binding protein (CREB), thereby controlling gene transcription [15,16,17]. During muscle development, studies have revealed that the *CaMK4* gene interacts with transcription factors or proteins in multiple muscle development-related signaling pathways [18]. In muscle tissue, *CaMK4* is involved not only in regulating cell proliferation but also closely linked to glucose metabolism, modulating insulin signaling to maintain energy homeostasis in muscle fibers [19,20]. Another study on mouse skeletal muscle revealed that overexpression of *CaMK4* significantly promotes mitochondrial biogenesis and fiber type conversion from fast-twitch to slow-twitch fibers, while simultaneously upregulating *PGC-1α* expression [21]. Although *CaMK4* is well studied in human and rodent systems, its role in ruminant muscle biology remains unexplored, hindering a comprehensive understanding of its regulatory role in muscle growth.

Multi-omics integration has helped reveal differentiation trajectories of muscle stem cells, such as satellite cells, during development and regeneration. For instance, integrating single-cell transcriptomic and spatial transcriptomic data have identified rare transitional progenitor states that are crucial for muscle regeneration [22]. Furthermore, combining epigenomic data (e.g., ATAC-Seq) with transcriptomics help elucidate how gene regulatory networks drive myogenesis [23].

Therefore, we used goat MuSCs as a model to systematically investigate the regulatory role of *CaMK4* in MuSCs proliferation using transcriptomic and proteomic approaches. *CaMK4* overexpression and knockdown models were established to assess its impact on cell proliferation and to identify key regulatory pathways and molecular networks. This study aims to fill the knowledge gap regarding the role of *CaMK4* in muscle development in ruminants, providing potential molecular insights and targets for muscle development research to enhance meat goat production.

## 2. Materials and Methods

### 2.1. Isolation, Culture, and Identification of Skeletal Muscle Satellite Cells

All animal procedures in this study conducted in strict accordance with the Guidelines for the Ethical Review of Laboratory Animal Welfare (GB/T 35892-2018) and were approved by the Southwest University Animal Ethics Committee (Approval No. IACUC-20240723-01, 25 July 2024). Fresh longissimus dorsi muscle tissue was collected from a healthy 1-month-old Dazu black goat, sourced from the Experimental Goat Farm of Southwest University (29°49′ N, 106°25′ E). Muscle satellite cells (MuSCs) were isolated using mechanical mincing combined with enzymatic digestion. Cells were purified by differential adhesion and cultured in complete DMEM medium supplemented with 10% fetal bovine serum and 1% penicillin-streptomycin at 37 °C under 5% CO_2_.

The purity and identity of MuSCs were verified by immunofluorescence staining using a PAX7 Polyclonal antibody (Proteintech, 20570-1-AP, Chicago, IL, USA), with nuclei counterstained by DAPI (Solarbio, Beijing, China). Nuclei appeared blue, while Pax7-positive cells showed green nuclear localization, confirming the MuSCs phenotype. Fluorescence images were captured using an inverted fluorescence microscope (Leica, Wetzlar, Germany).

### 2.2. Construction of CaMK4 Overexpression Lentiviral Vector and CaMK4-Targeted shRNA Interference Plasmid 

To investigate the role of *CaMK4* in muscle satellite cells (MuSCs), a *CaMK4* overexpression lentiviral vector and *CaMK4*-targeting shRNA plasmid were constructed. The *CaMK4* overexpression vector (HBLV-g-CAMK4-3XFLAG-ZsGreen-PURO) and *CAMK4*-targeted shRNA plasmid (pHBLV-U6-MCS-CMV-ZsGreen-PGK-PURO) were custom synthesized by Hanheng Biotechnology Co., Ltd. (Shanghai, China). Transfect cells at 50–70% confluence and observe fluorescence after 72 h of incubation. After transfection, total RNA was extracted using TRIzol reagent (Invitrogen, Carlsbad, CA, USA) according to the manufacturer’s instructions, and reverse-transcribed into cDNA using the PrimeScript RT reagent kit (Takara, Kyoto, Japan). Quantitative real-time PCR was performed with TB Green^®^ Premix Ex Taq™ II (Tli RNaseH Plus) (Takara, Japan) on a CFX96 Real-Time PCR Detection System (Bio-Rad, Hercules, CA, USA). *GAPDH* was used as the internal control, and relative gene expression was calculated using the 2^−ΔΔCt^ method. Primer sequences are listed in Supplementary Table S2 and were synthesized by Tianyihuiyuan Biotechnology Co., Ltd. (Wuhan, China).

### 2.3. Evaluation of CaMK4-Mediated Effects on Muscle Satellite Cell Proliferation and Cell Cycle Dynamics

To systematically investigate the role of *CaMK4* in goat muscle satellite cells (MuSCs), two treatment groups (four total groups) were established: *CaMK4* overexpression group (LV_OE_C) and its empty vector negative control (LV_NC_C); *CaMK4* knockdown group (LV_KD_S) and its corresponding empty vector control (LV_NC_S), each with three biological replicates, to ensure repeatability.

Cell Viability Assay (CCK-8): MuSCs were seeded in 96-well plates at a density of approximately 1 × 10^4^ cells/well. CCK-8 working solution (Solarbio, Beijing, China) was added at 24 h, 48 h and 72 h after transfection, and incubated for 2 h. Absorbance at 450 nm was measured using the Multiskan FC microplate reader (Thermo Fisher Scientific, Waltham, MA, USA) to assess changes in cell viability.

DNA Synthesis Assay (EdU): DNA synthesis was evaluated using the Cell-Light™ EdU Apollo^®^488 Stain Kit (Ribobio, C10371-3, Shanghai, China). Following 24-h transfection incubation, remove the virus-containing medium and add fresh complete medium. Continue culturing until 48 h, cells were incubated with 50 μM EdU solution for 3 h, followed by fixation, permeabilization, and staining according to the manufacturer’s protocol. Fluorescence images were captured with a fluorescence microscope (Leica, Germany), and the percentage of EdU-positive cells was quantified. Non-transfected cells served as negative controls to assess background signal and treatment effects on proliferation. Image analysis, including cell counting and signal intensity quantification, was performed using ImageJ(1.54j) software (National Institutes of Health, Bethesda, MD, USA), incorporating multi-channel image merging and EdU-positive cell percentage.

Cell Cycle Analysis: Transfected MuSCs were fixed overnight in 70% methanol, washed with PBS, and stained with FxCycle™ PI/RNase staining solution (Invitrogen, USA) for 15 min at room temperature in the dark. Cell cycle distribution (G0/G1, S, and G2/M phases) was analyzed by flow cytometry (BD FACSCanto II, Franklin Lakes, NJ, USA), and data were processed using FlowJo(v11) software (Stanford University, Palo Alto, CA, USA).

### 2.4. Transcriptomic Profiling Reveals CaMK4-Mediated Transcriptional Regulation in Goat Muscle Satellite Cells

To systematically investigate the transcriptional regulatory role of *CaMK4* in goat muscle satellite cells (MuSCs), this experiment established two treatment groups consistent with cellular function: LV_OE_C and LV_NC_C; LV_KD_S and LV_NC_S. Each group included four biological replicates to ensure reproducibility of the experimental results.

Following cell transfection, total RNA was extracted for subsequent transcriptome sequencing. Libraries were prepared using the NEBNext^®^ Ultra™ RNA Library Prep Kit (New England Biolabs, Ipswich, MA, USA) and sequenced on the Illumina NovaSeq 6000 platform with paired-end reads. Raw sequencing data were quality-controlled using fastp(0.18.0) to remove adapter contamination and low-quality reads [24], yielding high-quality clean reads. Clean reads were aligned to the goat reference genome (*Capra hircus* ARS1) using HISAT2(v2.1.0) [25]. Transcript assembly was performed with StringTie(v1.3.4) based on alignment results [26]. Gene expression levels were quantified using RSEM(1.2.19) [27], generating read count matrices. Differentially expressed genes (DEGs) were identified using the DESeq2(v1.20.0) package [28] with thresholds of |log_2_(Fold Change)| > log_2_(1.5) and *p*-value < 0.05. Functional enrichment analyses for Gene Ontology (GO) and Kyoto Encyclopedia of Genes and Genomes (KEGG) pathways were conducted via the MetaboAnalyst 6.0 online platform (https://www.metaboanalyst.ca, accessed on 13 February 2025). Bonferroni correction was applied to *p*-value to reduce the false-positive rate. Terms with adjusted *p*-value < 0.05 were considered significant enrichment signals among differentially expressed genes.

To validate the transcriptomic results, several significantly regulated genes from both *CaMK4* overexpression and knockdown groups were randomly selected for RT-qPCR verification (e.g., *C1QTNF3*, *NR4A3* and *CCDC80*). Primer sequences are provided in Supplementary Table S2.

### 2.5. Proteomic Profiling and Functional Analysis of CaMK4-Mediated Effects in Muscle Satellite Cells Using DIA Mass Spectrometry

Cell samples for proteomic analysis were prepared and grouped as described in Section 2.4. After transfection, collect cells by centrifugation, rapidly frozen in liquid nitrogen, and ground thoroughly. Lysis buffer was added for complete protein extraction. Samples were heated at 95 °C and 1000 rpm for 10 min, cooled, then digested with trypsin buffer at 37 °C and 500 rpm for 2 h. The digestion reaction was terminated, and peptides were purified using the iST kit (PreOmics, Germany). All samples were analyzed using the Orbitrap Lumos mass spectrometer (Thermo Fisher Scientific, MA, USA) connected to the EASY-nLC 1200 system via DIA (Data Independent Acquisition) technology for proteomics analysis. Raw data quality control was conducted with QuiC(v5) software(Biognosys, Zurich, Switzerland) [29] to ensure data integrity and reproducibility. Subsequently, DIA data were quantitatively analyzed with Spectronaut Pulsar(v17) software(Biognosys, Zurich, Switzerland) [30] based on a Data-Dependent Acquisition (DDA) spectral library. Only identifications with a false discovery rate (FDR) ≤ 1% at both protein and peptide levels were retained for further analysis. Differential expression was assessed using fold change (|FC| > 1.2) and *p* < 0.05. Functional annotation of differentially expressed proteins (DEPs) was performed using GO and KEGG databases. Bonferroni correction was applied to *p*-value to reduce the false-positive rate. Terms with adjusted *p*-value < 0.05 were considered significant enrichment signals among differentially expressed proteins.

### 2.6. Joint Multi-Omics Analysis Identifies Critical CaMK4-Regulated Genes and Pathways in Muscle Satellite Cells

To comprehensively integrate transcriptomic and proteomic expression changes and identify key downstream targets and functional pathways regulated by *CaMK4*, we performed joint analysis of transcriptome and proteome data from *CaMK4* overexpression and knockdown groups. DEGs and DEPs with significant changes in both overexpression and knockdown conditions were extracted for intersection analysis. Venn diagrams were generated using the online tool (https://www.omicshare.com/, accessed on 15 February 2025) to identify intersecting genes exhibiting significant alterations at both transcriptional and protein levels, termed interacted candidate genes (ICGs). Subsequently, GO functional annotation and KEGG pathway enrichment analyses were conducted on the ICGs.

### 2.7. Statistical Analysis of Experimental Data

All key experiments, including RT-qPCR, CCK-8, EdU assay, and cell cycle analysis, were performed with at least three independent biological replicates (n ≥ 3). Data are presented as the mean ± standard deviation (mean ± SD). Graphs and statistical analyses were generated using GraphPad Prism 9.0 (GraphPad Software, San Diego, CA, USA). Two-group comparisons were conducted using two-tailed unpaired Student’s *t*-test, with *p* < 0.05 considered statistically significant.

## 3. Results

### 3.1. Successful Construction of CaMK4 Overexpression and Knockdown Models

**Figure 1:**
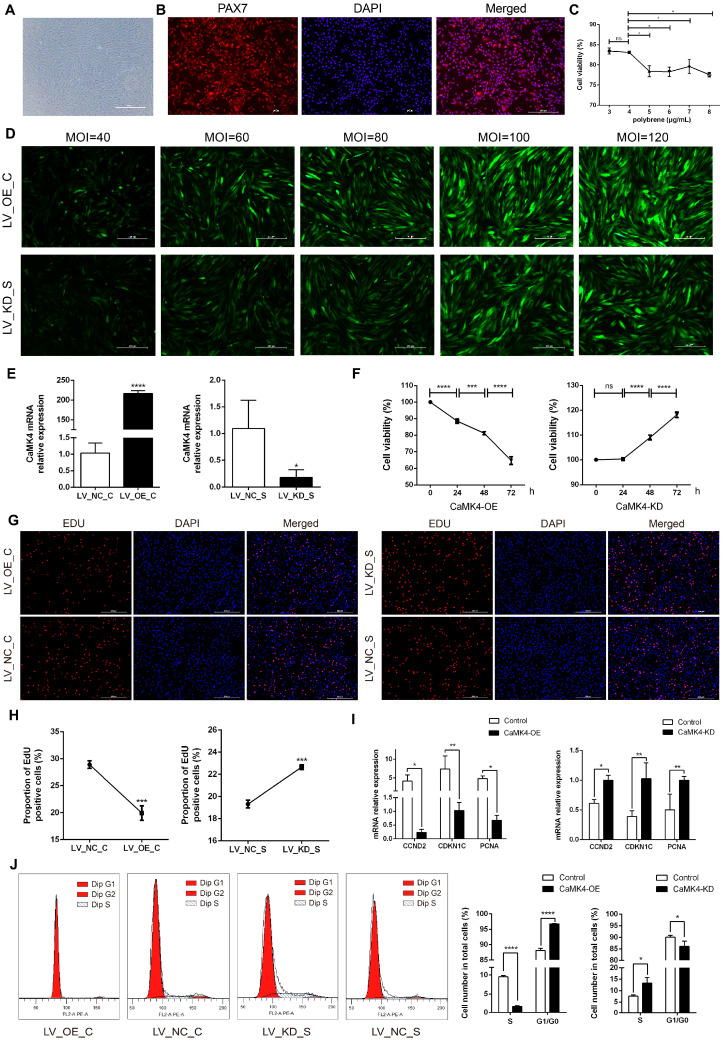
MuSCs activity, proliferation, and cycle detection. (**A**) Goat skeletal muscle satellite cells cultured. (**B**) Identification of goat MuSCs by immunofluorescence. Red fluorescence indicates PAX7 immunostaining, and blue indicates the nucleus. (**C**) Cell viability of MuSCs treated with gradient concentrations of polybrene (* *p* < 0.05, ns *p* > 0.05). (**D**) Detection of lentiviral transfection efficiency with gradient concentrations for *CaMK4* overexpression and knockdown. (**E**) Relative mRNA level of *CaMK4* after overexpression and knockdown treatments (* *p* < 0.05, **** *p* < 0.0001). (**F**) Cell activity of MuSCs measured by CCK-8 assay at different time points after *CaMK4* overexpression and knockdown (n = 3, *** *p* < 0.001, **** *p* < 0.0001, ns *p* > 0.05). (**G**) EdU assay detecting the proportion of proliferating MuSCs after *CaMK4* overexpression and knockdown. (**H**) Proportion of EdU-positive cells after *CaMK4* overexpression and knockdown (n = 3, *** *p* < 0.001). (**I**) Relative mRNA levels of proliferation marker genes after *CaMK4* gene overexpression and knockdown (* *p* < 0.05, ** *p* < 0.01). (**J**) Cell cycle analysis by flow cytometry after *CaMK4* overexpression and knockdown (n = 3, * *p* < 0.05, **** *p* < 0.0001).

Two hours after isolation and purification, goat skeletal muscle satellite cells (MuSCs) slowly adhered to the culture dish, exhibiting a typical elongated spindle shape (Figure 1A). Immunofluorescence detection showed that the positivity rate for PAX7 expression approached 100%, indicating that the purity of the isolated cells met the requirements for subsequent experiments (Figure 1B). After constructing the *CaMK4* overexpression plasmid and the *CaMK4*-shrNA interference plasmid, through a Polybrene concentration gradient experiment, 4 μg/mL was determined to be the optimal transfectant concentration, balancing transfection efficiency and cell viability (Figure 1C). Subsequently, lentiviral MOI gradient experiments showed that under MOI = 100 (overexpression group) and MOI = 80 (knockdown group) conditions, the cells exhibited the highest fluorescence intensity and normal morphology (Figure 1D). RT-qPCR detection further confirmed the significant transfection efficiency of both groups, ensuring the reliability of subsequent experimental data (Figure 1E).

### 3.2. CaMK4 Inhibits the Proliferation of Goat MuSCs

The results of CCK-8 cell viability assay (Figure 1F) indicated that the cell viability in the overexpression group decreased significantly over time (24, 48 and 72 h, *p* < 0.01), while that in the knockdown group showed a significant increase, suggesting that *CaMK4* was involved in inhibiting the proliferation process of MuSCs. As time increased, the survival rate for the overexpressed group decreased from 88.5% to 81.2% and then to 64.7%, while the survival rate for the control group increased from 100% to 109% and then to 118%. The EdU experiment verified the inhibitory effect of *CaMK4* on the proliferation of MuSCs (Figure 1G,H): overexpression of *CaMK4* significantly reduced the proportion of EdU-positive cells (1.45 times, *p* < 0.01), while it significantly increased in the knockdown group (1.17 times, *p* < 0.01). RT-qPCR analysis (Figure 1I) showed that the expressions of cell proliferation-related genes *CCND2*, *CDKN1C* and *PCNA* in the *CaMK4* overexpression group were significantly down-regulated (*p* < 0.05), while in the knockdown group, they showed a significant up-regulated trend. Flow cytometry analysis (Figure 1J) indicated that overexpression of *CaMK4* caused the cell cycle of MuSCs to arrest at the G0/G1 phase, from 88.1% to 96.7%, further supporting its function of inhibiting cell proliferation.

### 3.3. Study on the Transcriptional Regulatory Mechanisms of CaMK4 Overexpression

**Figure 2:**
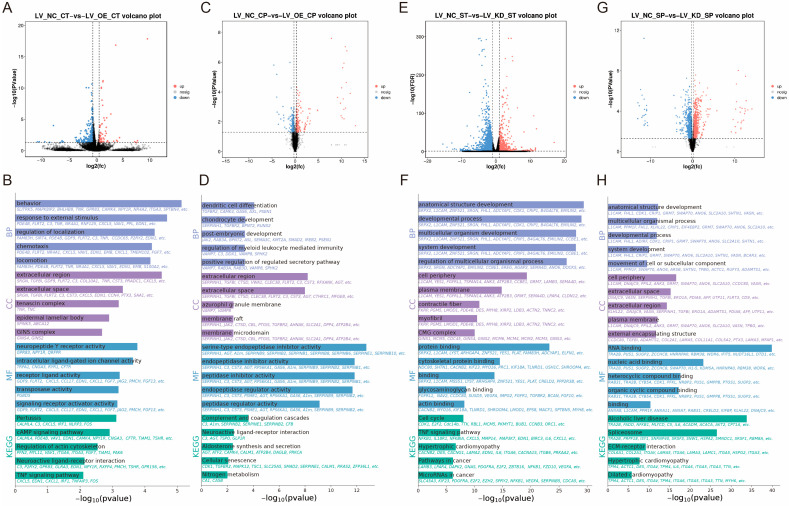
Research on the regulatory mechanisms of *CaMK4* overexpression, knockdown transcriptomics, and proteomics. (**A**) Differentially expressed genes after *CaMK4* overexpression. (**B**) KEGG pathway and GO term enrichment analysis of differentially expressed genes following *CaMK4* overexpression. (**C**) Differentially expressed proteins after *CaMK4* overexpression. (**D**) KEGG pathway and GO term enrichment analysis of differentially expressed proteins after *CaMK4* overexpression. (**E**) Differentially expressed genes after *CaMK4* knockdown. (**F**) KEGG pathway and GO term enrichment analysis of differentially expressed genes following *CaMK4* knockdown. (**G**) Differentially expressed proteins after *CaMK4* knockdown. (**H**) KEGG pathway and GO term enrichment analysis of differentially expressed proteins after *CaMK4* knockdown.

In this study, total cellular RNA was extracted from eight samples of LV_OE_CT and LV_NC_CT for transcriptomic sequencing. A total of 350 genes that exhibited significant DEGs were identified (Figure 2A), including 64 up-regulated genes and 286 down-regulated genes.

KEGG enrichment analysis (Figure 2B) revealed that a significant number of genes were enriched in the cAMP signaling pathway (e.g., *PDE4B*, *VAV1*, and *EDN1*), the regulation of the actin cytoskeleton (e.g., *PFN2*, *MYL11*, and *FGF7*), and the PI3K-Akt signaling pathway (e.g., *TNR*, *NR4A1*, and *FGF7*). These pathways have been associated with cell proliferation and muscle development. GO enrichment analysis (Figure 2B) demonstrated that a substantial proportion of entries were associated with DNA-binding transcription activator activity (e.g., *NR4A1*, *HOXD3*, and *MYBL1*), fibroblast growth factor receptor binding (e.g., *FLRT2* and *FGF7*), DNA binding (e.g., *NR4A1*, *MYBL1*, and *HOXD3*), and growth factor activity (e.g., *GDF9*, *FGF7*, and *JAG2*), among other biological functions related to cell proliferation. Furthermore, there are some GO entries related to signaling, such as signaling receptor activator activity (e.g., *GDF9*, *FLRT2*, and *CXCL5*) and ligand-gated calcium channel activity (e.g., *TRPA1* and *RYR1*). In summary, overexpression of *CaMK4* leads to a significant downregulation of the transcription activator *MYBL1* and the developmental regulator *HOXD3*.

### 3.4. Study on the Proteomic Regulatory Mechanisms of CaMK4 Overexpression

Among the 8 protein samples from LV_OE_CP and LV_NC_CP after *CaMK4* gene overexpression treatment, 446 DEPs were identified (Figure 2C), including 227 proteins that were upregulated and 219 proteins that were downregulated. KEGG enrichment analysis revealed (Figure 2D) that pathways including steroid hormone biosynthesis (e.g., COMT, PGFS, and HSD11B1), calcium signaling pathway (e.g., TNNC2, CA1, and CA5B), and JAK-STAT signaling pathway (e.g., JAK2, GFAP, and CCND2). Additionally, we found that the PCNA was enriched in signaling pathways related to cell proliferation and muscle development, such as DNA replication, mismatch repair, and cell cycle. GO enrichment analysis (Figure 2D) revealed numerous entries related to biological processes associated with DNA synthesis, such as DNA N-glycosylase activity (e.g., TDG and PCNA), histone binding (e.g., NCAPG2, JAK2, and APBB1), and MutLalpha complex binding (e.g., WRN and PCNA). Additionally, we found that the ACTN2 is enriched in GO entries related to cation binding, metal ion binding, ion binding, and calcium ion binding. In summary, *CaMK4* overexpression significantly reduces the protein abundance of molecules associated with cell proliferation and muscle development.

### 3.5. Study on the Transcriptional Regulatory Mechanisms of CaMK4 Knockdown

In this study, total cellular RNA was extracted from eight samples of LV_KD_ST and LV_NC_ST for transcriptomic sequencing. A total of 2525 DEGs were identified (Figure 2E), including 664 that were upregulated and 1861 that were downregulated. KEGG enrichment analysis (Figure 2F) revealed that many genes were significantly enriched in the cell cycle (e.g., *E2F2*, *CCND2*, and *TTK*), DNA replication (e.g., *FEN1*, *MCM5*, and *PCNA*), focal adhesion (e.g., *CCND2*, *MYL11*, and *PDGFRA*), and the regulation of the actin cytoskeleton (e.g., *CCND2*, *MYL11*, and *PDGFRA*). These pathways are related to muscle function and cell proliferation. GO enrichment analysis (Figure 2F) showed that a large number of entries related to muscle cell growth and development were significantly enriched, such as cytoskeletal protein binding (e.g., *SRPX2*, *L1CAM*, and *LYST*), actin binding (e.g., *CACNB2*, *MYO16*, and *KIF18A*), and tubulin binding (e.g., *NDC80*, *KIF23*, and *PRC1*). Notably, a large number of genes are enriched in GO entries related to cell proliferation, including binding (e.g., *SRPX2*, *GEM*, and *GSTM1*), DNA-binding transcription factor activity (e.g., *TBX1*, *FOXD1*, and *FOSB*), transcription regulatory region nucleic acid binding (e.g., *DACH1*, *TBX1*, and *CCAR1*), and protein binding (e.g., *SRPX2*, *GSTM1*, and *CCND2*). In summary, *CaMK4* knockdown significantly enhanced the expression of multiple genes associated with cell cycle regulation and cytoskeletal remodeling.

### 3.6. Study on the Proteomic Regulatory Mechanisms of CaMK4 Knockdown

Among the 8 protein samples from LV_KD_SP and LV_NC_SP after CaMK4 gene knockdown treatment, 1707 DEPs were screened (Figure 2G), of which 656 proteins were upregulated and 1051 proteins were downregulated. KEGG enrichment analysis revealed (Figure 2H) that DEPs were significantly enriched in ECM-receptor interaction (e.g., COL6A1, COL2A1, and LAMB2), DNA replication (e.g., RFC2, FEN1, and MCM5), focal adhesion (e.g., MYL11, LAMB2, and COL6A1), PI3K-Akt signaling pathway (e.g., JAK2, LAMB2, and COL6A1), and cardiac muscle contraction (e.g., TPM4, ACTC1, and ATP1B3), among other cell proliferation and muscle contraction-related signaling pathways. GO enrichment analysis (Figure 2H) showed that GO terms significantly related to cell proliferation, including RNA binding (e.g., TRA2B, SNRPD3, and FMR1), nucleic acid binding (e.g., TRA2B, PUS1, and SUGP2), and growth factor binding (e.g., VASN, NRP1, and COL2A1). In summary, *CaMK4* knockdown significantly increased the abundance of proteins associated with interactions with focal adhesions and the extracellular matrix (ECM).

### 3.7. Combined Analysis of CaMK4 Overexpression and Knockdown Transcriptomics and Proteomics

**Figure 3:**
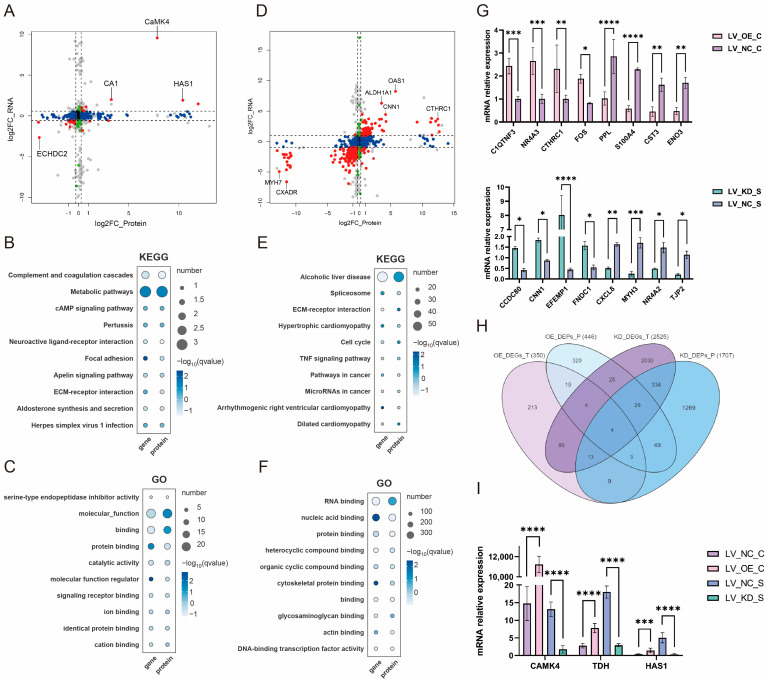
*CaMK4* overexpression and knockdown transcription-proteomics combined analysis. (**A**) Nine-quadrant plot of differential analysis of mRNAs and proteins after *CaMK4* overexpression. (**B**) KEGG pathway analysis of differentially expressed mRNAs and proteins after *CaMK4* overexpression. (**C**) GO term analysis of differentially expressed mRNAs and proteins after *CaMK4* overexpression. (**D**) Nine-quadrant plot of differential analysis of mRNAs and proteins after *CaMK4* knockdown. (**E**) KEGG pathway analysis of differentially expressed mRNAs and proteins after *CaMK4* knockdown. (**F**) GO term analysis of differentially expressed mRNAs and proteins after *CaMK4* knockdown. (**G**) RT-qPCR validation of differentially expressed genes after *CaMK4* overexpression and knockdown (* *p* < 0.05, ** *p* < 0.01, *** *p* < 0.001, **** *p* < 0.0001). (**H**) Venn diagram of DEGs and DEPs across the four comparisons. (**I**) Expression levels of the four intersecting genes and *CaMK4* from transcriptome sequencing data (*** *p* < 0.001, **** *p* < 0.0001).

Based on the results of transcriptional and protein differential analysis following *CaMK4* gene overexpression treatment, this study identified a total of 26 intersecting ICGs (e.g., *CA1*, *HAS1*, *CaMK4*, and *ECHDC2*) (Figure 3A). KEGG enrichment analysis results showed (Figure 3B) that *CA1* and *TDH* genes were enriched in metabolic pathways, nitrogen metabolism, and glycine, serine, and threonine metabolism, which are signaling pathways related to metabolic processes. GO enrichment analysis results (Figure 3C) show that a large number of genes (e.g., *TGFBI*, *C3*, and *TDH*) are enriched in GO terms related to molecular function regulators, binding, and protein binding.

Based on the results of transcriptional and protein differential analysis following *CaMK4* gene knockdown treatment, this study identified 377 intersecting ICGs (e.g., *OAS1*, *ALDH1A1*, *MYH7*, and *CXADR*) (Figure 3D). KEGG enrichment analysis results showed (Figure 3E) that the cell cycle (e.g., *PPIF*, *NFKB1*, and *DDIT3*), spliceosome (e.g., *NFKB1*, *HSP90B1*, and *TGFBR2*), P53 signaling pathway (e.g., *NFKB1*, *ARF6*, and *ABL2*), and calcium signaling pathway (e.g., *MYL11*, *ITGA6*, and *MYLK*), which are highly associated with cell proliferation processes, were significantly enriched. Notably, a large number of downregulated genes (e.g., *NFKB1*, *CSF1*, and *MAP2K1*) were enriched in the cell cycle and P53 signaling pathways. Additionally, pathways related to muscle tissue development, such as Regulation of actin cytoskeleton (e.g., *PPIF*, *NFKB1*, and *ABL2*) and Focal adhesion (e.g., *NFKB1*, *CTSK*, and *DDIT3*). GO enrichment analysis results (Figure 3F) showed that nucleic acid binding (e.g., *L1CAM*, *MYLK*, and *CNN1*), and protein binding (e.g., *L1CAM*, *MYLK*, and *CNN1*) were the most significantly enriched.

RT-qPCR results (Figure 3G) showed that the expression trends of the selected DEGs were consistent with the sequencing data, indicating that the transcription data analysis results in this part of the study were reliable. Further analysis of the 26 overlapping ICGs between the *CaMK4* gene overexpression group and the 377 overlapping ICGs in the *CaMK4* gene knockdown group revealed that four genes remained overlapping after the two groups were further intersected: *LGALS9*, *CTHRC1*, *HAS1*, and *TDH* (Figure 3H). Notably, *HAS1* and *TDH* were upregulated at both the mRNA and protein levels in the *CaMK4* gene overexpression group, while they were downregulated in the *CaMK4* gene knockdown group (Figure 3I).

## 4. Discussion

This study systematically revealed the important role of the *CaMK4* gene in regulating the proliferation of goat MuSCs. The results showed that *CaMK4* overexpression significantly inhibited MuSCs proliferation, as evidenced by decreased expression levels of the proliferation marker genes *PCNA*, *CCND2*, and *CDKN1C*; conversely, *CaMK4* knockdown upregulated the expression of these genes, thereby promoting cell proliferation. This trend aligns with the critical role of PCNA in DNA synthesis [31,32] and the functional roles of *CCND2* and *CDKN1C* in regulating cell cycle progression [33,34]. Further cell cycle analysis revealed that *CaMK4* overexpression led to cell accumulation in the G0/G1 phase, accompanied by a decrease in the proportion of cells in the S phase, suggesting that it inhibits cell proliferation by blocking the G1/S phase transition. Combining the known functional characteristics of calmodulin-dependent kinase in regulating cell cycle progression [35]. This study reveals that the *CaMK4*-mediated cell cycle arrest is an important mechanism limiting MuSCs proliferation, providing new theoretical basis for further understanding the molecular regulatory network of muscle growth and regeneration.

Additionally, it is important to emphasize that *CaMK4* exhibits significant functional heterogeneity across different cell types and species. For example, in human promyelocytic leukemia cells [36], mouse pancreatic β cells [37], and bone marrow hematopoietic stem cells [38], *CaMK4* exhibits an inhibitory effect on proliferation; whereas in mouse Tfh cells [39], renal cystic epithelial cells [40], and liver cancer cells [41], it promotes proliferation. This discrepancy may stem from variations in the cellular microenvironment, species specificity, and the activation status of upstream signaling pathways (such as MAPK/Bcl-2), suggesting that the regulatory mechanism of *CaMK4* in goat MuSCs has unique cell type-dependent characteristics.

Furthermore, as an important member of the calcium/calmodulin-dependent kinase family, *CaMK4* plays multiple roles in muscle development and functional regulation. It not only regulates skeletal muscle glucose metabolism and insulin gene expression but also promotes mitochondrial biogenesis and balances muscle energy metabolism [42,43]. At the molecular level, CaMK4 enhances the transcriptional activity of MEF2 by phosphorylating histone deacetylase 5 (HDAC5), thereby releasing the inhibitory complex formed between HDAC5 and the muscle-specific transcription factor MEF2. This promotes muscle gene expression and muscle cell differentiation [44,45,46]. This molecular mechanism provides a solid molecular basis for *CaMK4* regulation of muscle cell fate.

The transcriptomic and proteomic results of this study further reveal that *CaMK4* overexpression exerts a synergistic inhibitory effect on multiple signaling pathways associated with cell proliferation and muscle development. Specifically, *CaMK4* overexpression leads to a significant downregulation of the transcription activator *MYBL1* and the developmental regulator *HOXD3*. Previous studies have shown that MYBL1, as a potent transcription activator, promotes cell proliferation and signal transduction in various cell types [47,48], while miR-145-3p inhibition of *MYBL1* expression reduces MuSCs proliferation and mitochondrial function, highlighting its central role in proliferation regulation [49]. Similarly, high expression of *HOXD3* during development promotes cell proliferation, and its knockdown is associated with increased apoptosis [50]. Therefore, the synchronous downregulation of both suggests that *CaMK4* may weaken the proliferative potential of MuSCs by inhibiting the activity of key transcription factors at the transcriptional regulation level.

At the proteomic level, *CaMK4* overexpression significantly reduces the protein abundance of JAK2, ACTN2, and PCNA, which are associated with cell proliferation and muscle development. The JAK2 tyrosine kinase-mediated JAK2/STAT3 signaling pathway plays a key role in muscle cell proliferation and atrophy regulation [51,52], and its inhibition can induce muscle cell apoptosis [53]. ACTN2, as a skeletal muscle-specific actin structural protein, is closely associated with muscle weakness and muscle development abnormalities [54,55]. *PCNA* is an essential factor for cells to transition from the G1 phase to the S phase, participating in DNA replication and repair, and is also upregulated as a proliferation marker during muscle repair [56,57]. The significant downregulation of these proteins not only indicates that *CaMK4* exerts multi-level inhibition on MuSCs proliferation but also suggests that it may impair muscle tissue regeneration capacity after injury.

More importantly, joint analysis revealed that *TGFBI* was significantly upregulated at both the transcriptomic and proteomic levels, while *C3* was simultaneously downregulated. *TGFBI* is a potent inhibitory factor in skeletal muscle development, acting by blocking the PI3K/Akt/mTOR signaling pathway to inhibit muscle cell proliferation and differentiation [58,59]. In contrast, C3 participates in muscle remodeling by promoting the differentiation of myoblasts (including satellite cells) during muscle regeneration, and its downregulation suggests that the differentiation process may be impaired [60].

In contrast to the inhibitory effects of *CaMK4* overexpression, further analysis revealed that *CaMK4* knockdown significantly enhanced the expression of multiple genes associated with cell cycle regulation and cytoskeletal remodeling, such as *GSTM1* and *CCND2*. *GSTM1*, as a key molecule involved in oxidative stress metabolism, can influence cell activity and cycle progression by regulating the NF-κB and STAT3 signaling pathways [61,62]. *CCND2*, on the other hand, promotes G1/S phase transition, thereby directly enhancing cell proliferation capacity [63,64].

At the proteomic level, *CaMK4* knockdown significantly increased the abundance of proteins associated with interactions with focal adhesions and the extracellular matrix (ECM), such as laminin beta 2 (LAMB2) and type VI collagen alpha 1 (COL6A1). As is well known, focal adhesions are the core structural and signaling hubs connecting the cytoskeleton to the ECM, maintaining cell morphology while participating in signaling regulation [65,66]. LAMB2 activates cyclin D1 to promote entry into the S phase [67], while *COL6A1*, as an essential ECM component for MuSCs self-renewal and muscle regeneration, is critical for muscle tissue stability [68,69].

Further joint analysis of transcriptomic and proteomic data revealed that myosin light chain kinase (*MYLK*) and calmodulin subtype 1 (*CNN1*) were significantly upregulated at both the transcriptional and protein levels. As a member of the CAMKs family, MYLK enhances skeletal muscle sensitivity to Ca^2+^ and mechanical performance by regulating the phosphorylation level of myosin light chain [70,71]. Although *CNN1* is traditionally considered a smooth muscle marker protein, it also exhibits detectable expression levels in skeletal muscle, with functions closely associated with muscle cell proliferation and contraction. Previous studies have shown that *CNN1* knockdown inhibits the proliferation and differentiation of MuSCs [72,73]. Notably, a large number of downregulated genes (e.g., *NFKB1*, *CSF1,* and *MAP2K1*) are enriched in the cell cycle and p53 signaling pathways. In the classic p53-p21-RB axis, p53 can upregulate p21 expression by activating *CDKN1A*, thereby synergistically inhibiting the transcription of cell cycle genes with the RB-E2F complex, leading to G1 phase arrest and blocking the activation of S phase genes [74]. This study found that *CDKN1A* gene expression was downregulated, suggesting that *CaMK4* knockdown promotes cell proliferation by inhibiting the p53 signaling pathway. These data support the notion that *CaMK4* knockdown promotes the growth and development of MuSCs through skeletal structure regulation.

Additionally, this study found that *CaMK4* showed significant positive correlations with L-threonine dehydrogenase (*TDH*) and hyaluronan synthase 1 (*HAS1*) at both the transcriptional and protein levels. TDH, as the rate-limiting enzyme in mitochondrial threonine metabolism, mediates the conversion of threonine into glycine and acetyl-CoA, thereby linking energy metabolism and the tricarboxylic acid cycle [75]. In embryonic stem cells, *TDH* has been shown to regulate cellular activity and proliferation status; its inhibition induces autophagy and leads to proliferation arrest [76,77]. Meanwhile, HAS1 participates in the synthesis of hyaluronic acid (HA), a major component of the extracellular matrix (ECM). HA promotes the migration and proliferation of MuSCs during tissue development, injury repair, and muscle regeneration [78]. Although the HA synthesis rate of *HAS1* is lower than that of *HAS2*, its peak expression during early muscle differentiation suggests a potential role in regulating initial muscle development [79,80]. *HAS1* is significantly upregulated during muscle hypertrophy. Studies indicate that following Achilles tendon resection-induced plantar muscle hypertrophy in rats, both *HAS1* gene expression and hyaluronic acid (HA) synthesis are markedly increased [81]. Overall, these results suggest that *CaMK4* participates in the co-regulation network of the metabolic microenvironment of MuSCs by regulating the *TDH*-mediated metabolic pathway and the ECM microenvironment regulated by *HAS1*. This influences the proliferation and function of MuSCs.

This study reveals the potential role of *CaMK4* in the proliferation of goat muscle-derived stem cells (MuSCs) and its underlying molecular mechanisms. These findings may provide new insights into goat muscle development and positively impact the production of high-quality meat goats. Additionally, they offer a reference for identifying candidate genes for cross-species genetic marker selection in subsequent domestic animal breeding programs. However, this study still has some limitations. First, the experiments were based primarily on in vitro systems and lacked in vivo validation. Second, the precise signaling pathways and upstream regulatory factors remain unclear. Future studies could combine gene editing with animal models to elucidate the biological functions of *CaMK4* further and utilize single-cell omics technologies to explore its dynamic roles in different muscle cell populations and microenvironments. This research would reveal the fine-tuned regulatory mechanisms of *CaMK4* and provide important theoretical foundations for improving meat performance in ruminants and for preventing and treating muscle-related diseases.

## 5. Conclusions

This study clearly demonstrated that *CaMK4* acts as a negative regulator of goat MuSC proliferation by suppressing key genes and proteins involved in cell cycle progression, cytoskeletal organization, and muscle development. Knockdown of *CaMK4* promoted cell adhesion- and ECM-related pathways, as well as metabolic processes such as threonine metabolism, thereby enhancing skeletal muscle growth potential. By integrating transcriptomic and proteomic data, we established a molecular framework for *CaMK4*-mediated regulation in muscle development. These findings may not only advance our understanding of muscle growth mechanisms in ruminants but also offer new insights for improving meat quality and production efficiency. Future work will combine gene editing with animal models to further elucidate specific signaling pathways and upstream regulatory factors.

## Data Availability

The bulk RNA-seq data generated from study are available from SRA database (PRJNA1228310: SRR32638629-44). The proteome sequencing data generated in this study can be obtained from the iProX database (PXD067535).

## Supplementary Materials

The following supporting information can be downloaded at: https://www.mdpi.com/article/10.3390/ani15213083/s1. Table S1: The viral vector siRNA sequences and shRNA sequence information; Table S2: Primer sequences for cell proliferation and transcriptome validation genes; Table S3: Transcriptomic DEGs upon *CaMK4* overexpression in goat MUSCs; Table S4: GO enrichment analysis of transcriptomic DEGs upon *CaMK4* overexpression in goat MUSCs; Table S5: KEGG enrichment analysis of transcriptomic DEGs upon *CaMK4* overexpression in goat MUSCs; Table S6: Proteomic DEPs upon *CaMK4* overexpression in goat MUSCs; Table S7: GO enrichment analysis of proteomic DEPs upon *CaMK4* overexpression in goat MUSCs; Table S8: KEGG enrichment analysis of proteomic DEPs upon *CaMK4* overexpression in goat MUSCs; Table S9: Transcriptomic DEGs upon *CaMK4* knockdown in goat MUSCs; Table S10: GO enrichment analysis of transcriptomic DEGs upon *CaMK4* knockdown in goat MUSCs; Table S11: KEGG enrichment analysis of transcriptomic DEGs upon *CaMK4* knockdown in goat MUSCs; Table S12: Proteomic DEPs upon *CaMK4* knockdown in goat MUSCs; Table S13: GO enrichment analysis of proteomic DEPs upon *CaMK4* knockdown in goat MUSCs; Table S14: KEGG enrichment analysis of proteomic DEPs upon *CaMK4* knockdown in goat MUSCs; Table S15: GO enrichment analysis of DEGs from joint transcriptomic and proteomic analysis upon *CaMK4* overexpression in goat MUSCs; Table S16: KEGG enrichment analysis of DEGs from joint transcriptomic and proteomic analysis upon *CaMK4* overexpression in goat MUSCs; Table S17: KEGG enrichment analysis of DEGs from joint transcriptomic and proteomic analysis upon *CaMK4* knockdown in goat MUSCs; Table S18: GO enrichment analysis of DEGs from joint transcriptomic and proteomic analysis upon *CaMK4* knockdown in goat MUSCs.
